# Self-esteem and appearance anxiety among Chinese college students: the roles of social media use and upward social comparison

**DOI:** 10.3389/fpsyg.2025.1562711

**Published:** 2025-06-02

**Authors:** Anqi Shang, Han Bao

**Affiliations:** Department of Media and Communication, Yeungnam University, Gyeongsan, Republic of Korea

**Keywords:** self-esteem, appearance anxiety, social media, upward social comparison, gender differences

## Abstract

This study aimed to examine the mechanisms underlying the association between self-esteem and appearance anxiety, and whether these mechanisms differ by gender. Using structural equation modeling (SEM), we investigated interrelationships among self-esteem, social media use, upward social comparison, and appearance anxiety in a sample of 487 college students (52% female) from Jilin and Shanxi provinces, China. Our analysis showed a significant negative correlation between self-esteem and appearance anxiety. This association was partially explained by social media use and a tendency to make upward social comparisons, and this indirect effect was found to be moderated by gender. Specifically, among males, making upward social comparisons was not associated with appearance anxiety. Our findings provide insights into the link between low self-esteem and appearance anxiety in an understudied population.

## 1 Introduction

Social media is awash with content posted by individuals dissatisfied with their appearance, with many using these platforms to criticize their appearance, describe their anxieties, and express a desire for ideal beauty. Appearance anxiety is commonly defined as a form of stress experienced in response to others’ evaluations of one’s physical appearance (Hart et al., 2008). A report on China’s medical aesthetics industry from a global data intelligence platform shows that 77% of Chinese citizens have experienced appearance anxiety, driving China’s medical aesthetics market to grow by 105% between 2017 and 2022 (Mob-Tech Research Institute, 2023). The adverse effects of appearance anxiety can manifest as eating disorders (Levinson et al., 2013), body dysmorphic disorder (Veale et al., 2014), body dissatisfaction and shame (Gerrard et al., 2021; Markham et al., 2005), decreased self-efficacy (Iancu et al., 2015), increased loneliness (Sübaşı, 2010), and nomophobia, the fear of being without mobile phone connectivity (Ayar et al., 2018).

In non-Western and particularly collectivist societies, body image tends to be shaped not only by individual perceptions but also by socially shared standards and interpersonal expectations (Parihar et al., 2022; Grogan, 2021). Against this cultural backdrop, Chinese college students are a population particularly sensitive to appearance-related concerns (Jackson and Chen, 2008a; Chen et al., 2007). The prevalence of body dissatisfaction is rising among young people and college students in China (Chen et al., 2007; Xu et al., 2010). A survey conducted by Cheng and Ma (2021) revealed that appearance anxiety was reported by 60% of female college students and 37% of male students. Appearance anxiety among Chinese college students is associated with social anxiety (Liao et al., 2023), restrained eating behavior (Fu et al., 2022), and self-objectification (Jackson et al., 2016). However, evidence regarding the development of appearance anxiety among young people remains limited. Given the detrimental effects of appearance anxiety, further investigation into its causes and consequences is needed.

A useful lens to understand the development of appearance anxiety is social comparison theory (Festinger, 1954), which suggests that individuals evaluate themselves based on comparisons with others. In the context of social media, users are frequently exposed to curated images and idealized portrayals of others (Fardouly and Vartanian, 2016; Hawes et al., 2020; Monro and Huon, 2005), making upward social comparison—comparing oneself to those perceived as better (Vogel et al., 2014)—both common and impactful. Self-esteem, in particular, plays a foundational role in this process: individuals with lower self-esteem are more likely to engage in frequent social media use and upward social comparisons (Malik and Khan, 2015; Buunk and Gibbons, 2007). In light of this pattern, appearance anxiety may be statistically associated with patterns involving self-esteem, social media use, and upward social comparison. Therefore, this study draws on social comparison theory to examine these associations in a sample of Chinese college students.

Prior research has consistently shown that gender differences exist in appearance-related concerns and body image outcomes. For instance, females are more likely to engage in appearance-based social comparisons and report higher levels of body dissatisfaction than males (Lindberg et al., 2006; Myers and Crowther, 2009). Given these gendered tendencies, the present study aims to explore whether the hypothesized pathways function similarly across male and female college students.

### 1.1 Self-esteem and appearance anxiety

Self-esteem, an aspect of the self-concept, comprises both temporary and long-term subjective assessments of one’s worth (MacDonald and Leary, 2012). It is thought to be a relatively stable trait (Rosenberg, 2015). Although trait self-esteem is considered relatively stable over time (Orth and Robins, 2014), it is not entirely immune to contextual influences. Individuals may exhibit situational susceptibility in self-worth evaluations, particularly in socially comparative environments (Deci and Ryan, 1995). Therefore, in the current study, self-esteem is treated as a trait-like variable, but its association with social media use and upward comparison reflects susceptibility to contextual influences, rather than state-level fluctuations. Self-esteem reflects a sense of being “good enough” (Mecca et al., 1989), which is informed by perceptions rather than objective reality (Baumeister et al., 2003). The feelings that arise from such self-evaluations affect self-presentation, intimacy, emotional experiences, and responses to negative evaluations (Baumeister et al., 1989; Brown, 2010; Leary and Baumeister, 2000). Individuals with high self-esteem are less distressed by negative feedback compared to those with low self-esteem (Brown, 1986). Promoting self-esteem and preventing low self-esteem are acknowledged as important societal objectives (Orth and Robins, 2014).

Appearance anxiety is influenced by low self-esteem. Individuals experiencing this form of anxiety feel dissatisfaction with their physical appearance or body image, with this perceived imperfection causing worry about negative judgment from others (Hart et al., 2008). Self-esteem appears to serve as a barrier (Pyszczynski et al., 2004), protecting individuals from negative feedback and evaluations (Brown, 2010). Previous studies show that low self-esteem influences dissatisfaction and anxiety around one’s appearance (Frost and McKelvie, 2004; Kostanski and Gullone, 1998; Mellor et al., 2010). Adolescents with low self-esteem experience increased anxiety about their social appearance (Sahin et al., 2014). Accordingly, we propose the following:

*H1*: Self-esteem is negatively correlated with appearance anxiety.

### 1.2 Social media use as an indirect influencing factor

Industry reports estimate there are almost five billion users of social media worldwide, nearly 60% of the global population (We Are Social, 2023). Many use social media to construct an identity (Manago et al., 2008), with resulting implications for lifestyle, mental health, and life satisfaction (Coyne et al., 2020; Hawi and Samaha, 2017). In the Chinese context, college students frequently use platforms such as WeChat, Xiaohongshu (RED), Weibo, and Douyin. These platforms differ in their functions and visual formats, ranging from messaging to image-based social sharing. Prior studies have noted that self-presentation, entertainment, and information-seeking are among the most common purposes for social media use among Chinese youth (Gan and Wang, 2015; Ku et al., 2013). According to a report by Bian et al. (2024), nearly 90% of surveyed college students use social media to share diverse aspects of their lives. Despite this high usage rate and variety of purposes, cultural factors appear to influence how students engage with social media. Existing research suggests that Chinese college students often engage in social media use with greater social concern and hesitation, placing more emphasis on social evaluation and group harmony, in contrast to Western users, who are more commonly driven by self-enhancement motivations (Xu et al., 2018; Chu and Choi, 2010). Such purposes may be associated with a higher likelihood of engaging in appearance-based comparisons, which are, in turn, related to elevated appearance anxiety.

Self-esteem influences how individuals use social media. Users with low self-esteem are more likely to view social media as a safe platform for self-expression, as it reduces the perceived risks of self-expression (Forest and Wood, 2012). Consequently, these individuals tend to prefer online communication over face-to-face interactions (Andreassen et al., 2017). Self-esteem is negatively correlated with problematic use of social media (Malik and Khan, 2015). As such, we propose:

*H2*: Self-esteem is negatively correlated with social media use.

Social media is believed to influence young people’s concerns about their appearance due to platforms’ frequent depictions of idealized body images (Fardouly and Vartanian, 2016; Hawes et al., 2020; Monro and Huon, 2005). Portrayals of beauty standards on social media are often unrealistic and difficult to attain in a healthy manner (Lang et al., 2023). Exposure to these unrealistic beauty standards can lead individuals to internalize such ideals (Vandenbosch and Eggermont, 2012), increasing body dissatisfaction and social appearance anxiety (Groesz et al., 2002; Caner et al., 2022). It is reported that the widespread use of social media has exacerbated body image issues (Franchina and Lo Coco, 2018). As such, we predict that:

*H3*: Social media use is positively correlated with appearance anxiety.

*H4*: Social media use partially explains the association between self-esteem and appearance anxiety.

### 1.3 Upward social comparison as an indirect influencing factor

Social comparison theory (Festinger, 1954) posits that individuals evaluate their worth and abilities by comparing themselves to others. Upward social comparison refers to comparing oneself to individuals perceived as more successful (Vogel et al., 2014). Engaging in upward social comparisons often leads to self-disapproval and denial, resulting in low self-evaluation and negative emotions (Collins, 1996; Schmuck et al., 2019; Wheeler and Miyake, 1992). Such comparisons can trigger anxiety and feelings of inadequacy, reducing subjective well-being (Wheeler and Miyake, 1992). Individuals with low self-esteem often have an unstable self-concept, encouraging upward social comparisons (Buunk and Gibbons, 2007; Wheeler and Miyake, 1992). As such, self-esteem is negatively correlated with upward social comparison (Schmuck et al., 2019). In a study of college students, Vohs and Heatherton (2004) found that when self-image was threatened, students with high self-esteem were more likely to make downward social comparisons as a self-protection strategy, whereas those with low self-esteem made upward social comparisons. The pursuit of self-improvement through upward social comparisons may explain why those with low self-esteem habitually engage in such comparisons (Ybema and Buunk, 1993). We therefore propose:

*H5*: Self-esteem is negatively correlated with upward social comparison.

Upward social comparisons are linked to general anxiety and state anxiety (McCarthy and Morina, 2020; Zheng et al., 2020). When comparisons focus on appearance, they can contribute to body dissatisfaction (Thøgersen-Ntoumani et al., 2017), body image avoidance (Rodgers et al., 2013), eating disorders (Holland and Tiggemann, 2016), and numerous appearance-related anxiety issues. Lin and Kulik (2002) found that exposure to images of slim peers resulted in decreased body satisfaction and confidence, and elevated anxiety. Similarly, Tiggemann and McGill (2004) reported experimental evidence that individuals who made social comparisons upon viewing images of idealized models experienced heightened negative affect, body dissatisfaction, and weight-related anxiety. We thus propose the following:

*H6*: Upward social comparison is positively correlated with appearance anxiety.

*H7*: Upward social comparison partially explains the association between self-esteem and appearance anxiety.

### 1.4 Social media use and upward social comparison

Social media platforms encourage meticulous self-presentation, facilitate extensive social comparisons, and expose individuals to a constant flow of feedback (Madden et al., 2013). Sharing personal information and social interactions on social media are common triggers for social comparisons (Lup et al., 2015). The information shared on social media is often idealized and retouched (Vandenbosch and Eggermont, 2012), likely impelling upward social comparison, particularly among those with low self-esteem. Therefore, we put forth the following:

*H8*: Social media use is positively correlated with upward social comparison.

*H9*: There is a chain indirect effect from self-esteem to appearance anxiety via social media use and upward social comparison.

**Figure 1:**
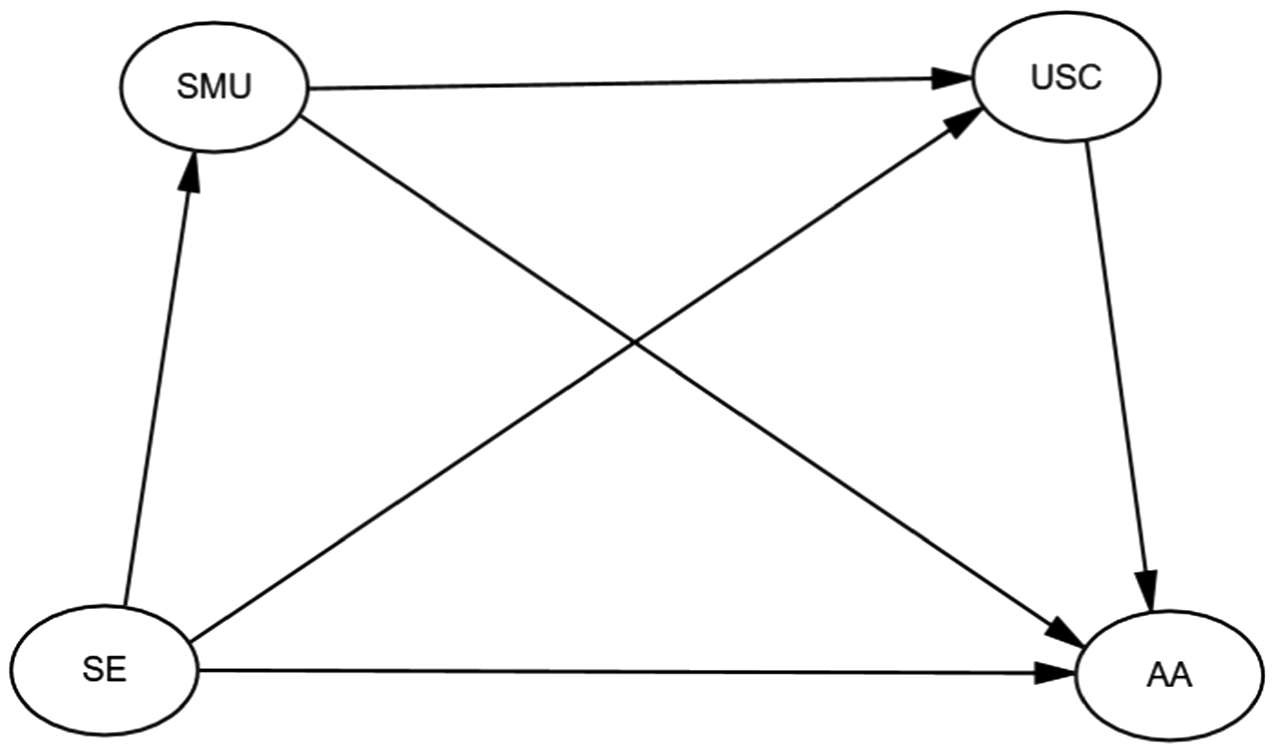
Conceptual research model. SE, self-esteem; SMU, social media use; USC, upward social comparison; AA, appearance anxiety.

To examine the associations among self-esteem, social media use, upward social comparison, and appearance anxiety, the present study proposes a conceptual framework informed by social comparison theory (Festinger, 1954). Figure 1 presents the hypothesized model, which illustrates potential associations between self-esteem and appearance anxiety, both directly and indirectly via social media use and upward social comparison. In addition, gender was included in the model to explore potential moderation effects on the structural pathways.

## 2 Methods

### 2.1 Participants and procedure

This cross-sectional study was conducted online from January to March 2024, targeting college students from Jilin and Shanxi provinces in China. Participants were recruited using convenience sampling via university platforms and student WeChat groups. The inclusion criteria required that participants be currently enrolled undergraduate students between the ages of 18 and 25 who voluntarily agreed to participate. Before beginning the questionnaire, all participants were required to read an online informed consent form that explained the purpose of the study, the voluntary nature of participation, and the guarantee of anonymity. Only those who clicked “agree” were allowed to proceed to the survey. Throughout the data collection process, we adhered to standard ethical principles, including informed consent, voluntary participation, confidentiality of data, and informing participants that they could withdraw from the questionnaire at any time. Furthermore, of the 548 students who participated in the survey, 487 valid responses (88.9%) were retained after excluding 61 invalid ones (e.g., insufficient data, incorrect personal information, and identical response patterns). This sample size exceeds the commonly recommended threshold for structural equation modeling, which suggests having 10 to 20 participants per estimated parameter (Kline, 2023), and is consistent with empirical recommendations for models with moderate complexity (Wolf et al., 2013). The final sample consisted of 236 males and 251 females. Table 1 presents participants’ demographic information.

**Table 1:** Participant characteristics.

Variable	M	%
Total	487	
Gender		
Male	236	48.4
Female	251	51.6
Area		
Rural	255	52.4
Urban	232	47.6
Grade		
Freshman	114	23.4
Sophomore	127	26.1
Junior	110	22.6
Senior	136	27.9
Major		
Liberal arts	161	33.1
Science	152	31.2
Art and sport	174	35.7

### 2.2 Measures

#### 2.2.1 Self-esteem

We used the 10-item Self-Esteem Scale (Rosenberg, 1965), which has been widely used among Chinese student populations and has demonstrated good psychometric properties in multiple studies (Leung and Wong, 2008; Yu et al., 2022). Example items include “I consider myself a valuable person, at least on par with others” and “I think I have a lot going for me.” Participants responded on a four-point scale (1 = *totally disagree*, 4 = *totally agree*), with five of the items reverse-scored. Higher total scores indicate higher self-esteem. Internal consistency was excellent (*α* = 0.92).

#### 2.2.2 Social media use

Given the widespread use of social media platforms such as WeChat and Weibo among Chinese college students, we culturally adapted the Facebook Intensity Scale (Ellison et al., 2007) by replacing the term “Facebook” with the more general term “social media” to better suit the local context. This adapted scale has been widely employed in studies targeting Chinese university students and has demonstrated good psychometric properties in similar populations (Hong et al., 2014; Liu et al., 2013). The adapted scale consists of six items and is used to measure the intensity of social media use. Example items include “Social media is part of my everyday activity” and “I would be sorry if social media shut down.” Participants rated their agreement with each statement on a five-point scale (1 = *strongly disagree*, 5 = *strongly agree*). Higher total scores indicate higher intensity of social media use. Internal consistency was good (*α* = 0.85).

#### 2.2.3 Upward social comparison

We used the Chinese version of the Upward Social Comparison Scale (Bai et al., 2013), adapted from the Iowa-Netherlands Comparison Orientation Measure (Gibbons and Buunk, 1999). The scale has six items. Examples are “I often compare myself to people who are better than me” and “I often compare myself to people who are more successful than me.” Participants rated their agreement with each statement on a five-point scale (1 = *strongly disagree*, 5 = *strongly agree*). Higher total scores indicate a greater tendency to make upward social comparisons. The internal consistency of this measure was good (*α* = 0.88).

#### 2.2.4 Appearance anxiety

We used the 10-item Appearance Anxiety Inventory (Veale et al., 2014), which has demonstrated good cross-cultural reliability and validity in previous studies (Chan et al., 2024; Burkauskas et al., 2022). Example items are “I compare aspects of my appearance to others” and “I think about how to camouflage or alter my appearance.” Participants rated their agreement with each statement on a five-point scale (1 = *totally disagree*, 5 = *totally agree*). Higher total scores indicate higher appearance anxiety. Internal consistency was excellent (*α* = 0.95).

### 2.3 Data analysis

We used SPSS 20.0 for descriptive statistics, correlation analysis, and reliability testing, and AMOS 21.0 to assess the validity of measurement instruments, and conduct confirmatory factor analyses (CFAs), structural equation modeling, and multi-group comparisons. Before testing the full path model, separate CFAs were conducted for each latent construct (self-esteem, social media use, upward social comparison, and appearance anxiety) to confirm the measurement structure. Model fit was evaluated using multiple fit indices, including CFI, TLI, RMSEA, and SRMR. Composite reliability (CR) for each construct was computed based on standardized factor loadings and error variances from the CFA results. To assess direct and indirect effects, we employed the bias-corrected percentile bootstrap method with 5,000 resamples and 95% confidence intervals. An indirect effect was considered statistically significant when the confidence interval did not include zero.

## 3 Results

### 3.1 Preliminary analyses

#### 3.1.1 Common method deviation test

Exploratory factor analysis was used to test for possible common method biases. The findings revealed that four factors had eigenroot values exceeding 1. The first common factor accounted for 39.4% of the overall variance, which is below the recommended cutoff of 40% (Podsakoff et al., 2003). This suggests the data were not affected by common method bias.

#### 3.1.2 Correlations and descriptive tests

Table 2 shows the relations between the four main variables. Self-esteem was significantly negatively correlated with social media use (*r* = −0.16, *p* < 0.05), upward social comparison (*r* = −0.35, *p* < 0.05), and appearance anxiety (*r* = −0.69, *p* < 0.05). Social media use was significantly positively correlated with upward social comparison (*r* = 0.47, *p* < 0.05) and appearance anxiety (*r* = 0.38, *p* < 0.05). Upward social comparison was significantly positively correlated with appearance anxiety (*r* = 0.38, *p* < 0.05). The observed correlations supported further hypothesis testing.

**Table 2:** Descriptive and correlation analysis.

	1	2	3	4
Self-esteem	—			
Social media use	−0.16^**^	—		
Upward social comparison	−0.35^**^	0.47^**^	—	
Appearance anxiety	−0.69^**^	0.38^**^	0.52^**^	—
M	30.08	21.32	20.31	29.03
SD	5.88	4.80	4.90	9.73

^**^p < 0.01.

As shown in Table 2, participants’ scores on the four key variables exhibited a moderately high trend overall, indicating a certain degree of prevalence of these psychological characteristics within the sample. This pattern is generally consistent with findings from previous studies on university students (Chen et al., 2007; Xu et al., 2010; Franchina and Lo Coco, 2018).

At the same time, the sample demonstrated relatively active social media use and a marked tendency toward upward social comparison, accompanied by a certain level of appearance anxiety. This combination of characteristics reflects variable patterns consistent with the conceptual model of the present study, suggesting the possible existence of a potential indirect association between social media use, upward social comparison, and the relationship between self-esteem and appearance anxiety, and highlights how the observed sample characteristics may serve as a basis for future research on the associations among these variables.

### 3.2 Test of measurement model

**Figure 2:**
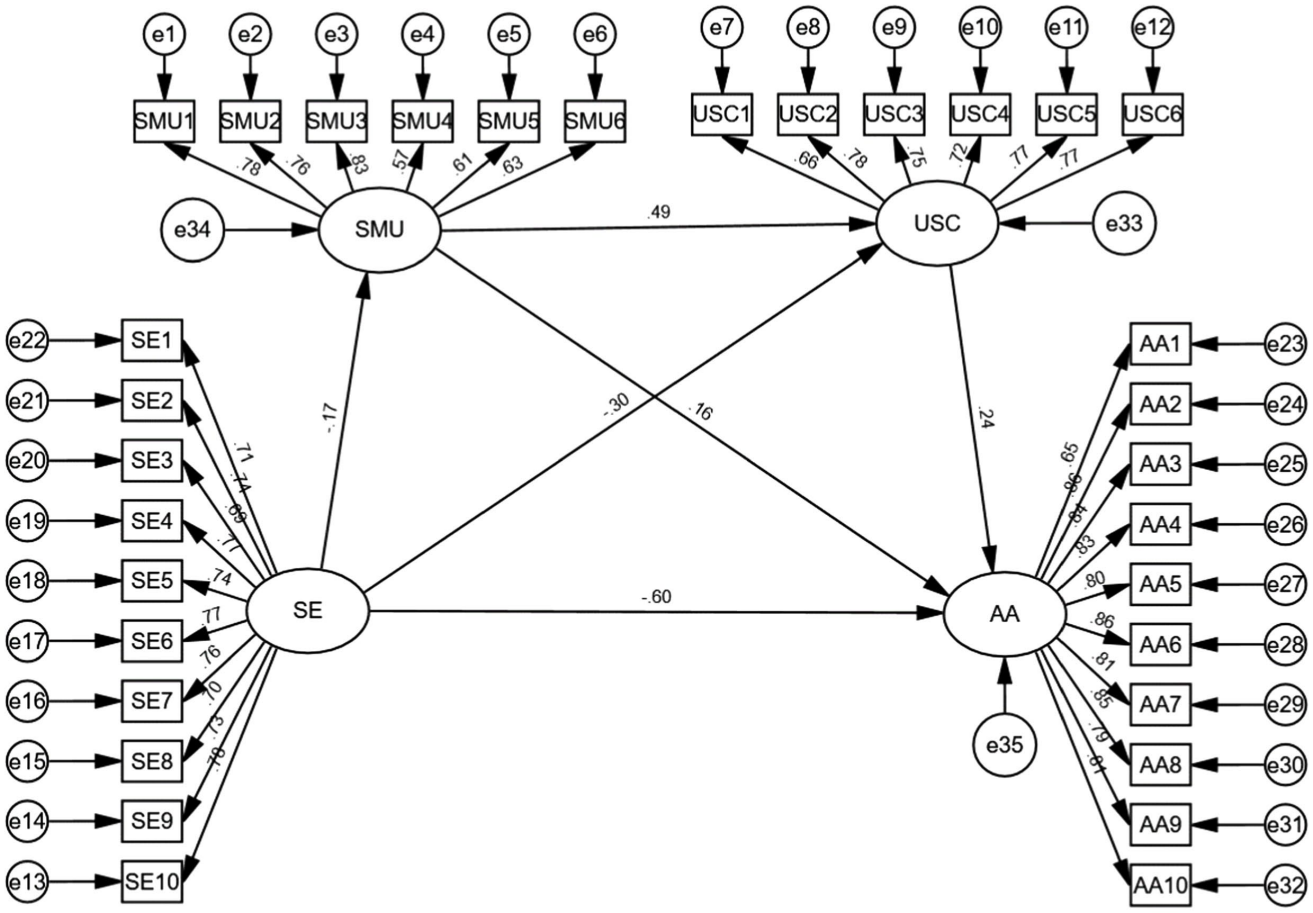
Standardized factor loadings and path coefficients in measurement model. SE, self-esteem; SMU, social media use; USC, upward social comparison; AA, appearance anxiety.

Figure 2 shows that all observed indicators in the measurement model significantly loaded on their hypothesized constructs. All standardized loads were >0.50 and meet the standard (Kline, 2023). Regarding model fit, most indices met recommended criteria (CMIN/df  = 2.86, *p* < 0.001, CFI = 0.92, IFI = 0.92, TLI = 0.91, PNFI = 0.81, PCFI = 0.83, RMSEA = 0.06; recommended criteria: CMIN/df <3, CFI, IFI/TLI, PCFI ≥0.90, RMSEA <0.80; Kline, 2023).

Table 3 shows that composite reliability values exceeded the recommended cutoff of 0.7 for all constructs. AVE values exceeded the cutoff score of 0.5 for all constructs except for social media use, for which AVE was slightly below the cut-off (0.49). However, this is considered acceptable as its composite reliability (CR = 0.85) exceeds the recommended threshold of 0.60, and the overall model demonstrated satisfactory convergent validity and reliability (Fornell and Larcker, 1981).

**Table 3:** Composite reliability and average variance extracted.

Variable	CR	AVE
Self-esteem	0.92	0.55
Social media use	0.85	0.49
Upward social comparison	0.88	0.55
Appearance anxiety	0.95	0.66

CR, composite reliability; AVE, average variance extracted.

Table 4 shows that correlations between constructs were all lower than the square root of the AVE, suggesting adequate discriminant validity (Fornell and Wernerfelt, 1987).

**Table 4:** Discriminant validity tests.

Variable	AVE	1	2	3	4
Self-esteem	0.55	0.74			
Social media use	0.49	−0.17	0.70		
Upward social comparison	0.55	−0.38	0.54	0.74	
Appearance anxiety	0.66	−0.72	0.39	0.55	0.81

### 3.3 Path analysis

#### 3.3.1 Test of direct effect

Direct effects in the model are presented in Table 5. Self-esteem was significantly negatively associated with appearance anxiety (*β* = −0.60, *p* < 0.001), supporting *H1*. Self-esteem was significantly negatively associated with social media use (*β* = −0.17, *p* < 0.001), supporting *H2*. Social media use was significantly positively associated with appearance anxiety (*β* = 0.16, *p* < 0.001), supporting *H3*. Self-esteem was significantly negatively associated with upward social comparison (*β* = −0.30, *p* < 0.001), supporting *H5*. Upward social comparison was significantly positively associated with appearance anxiety (*β* = 0.24, *p* < 0.001), supporting *H6*. Social media use was significantly positively associated with upward social comparison (*β* = 0.49, *p* < 0.001), supporting *H8*.

**Table 5:** Standardized direct effects.

Path	Estimate	SE	CR	*p*
Self-esteem → appearance anxiety	−0.60	0.05	−11.15	^***^
Self-esteem → social media use	−0.17	0.06	−3.30	^***^
Self-esteem → upward social comparison	−0.30	0.04	−6.37	^***^
Social media use → upward social comparison	0.49	0.05	9.05	^***^
Social media use → appearance anxiety	0.16	0.04	3.69	^***^
Upward social comparison → appearance anxiety	0.24	0.05	4.96	^***^

^***^p < 0.001.

#### 3.3.2 Test of indirect effect

An indirect effect was tested based on significant coefficients of direct paths. We used the bias-corrected percentile bootstrap method to test a chain mediation effect from self-esteem to appearance anxiety, via all the above-mediated paths. We selected a bootstrap self-sampling size of 5,000 and calculated 95% confidence intervals. Findings from this analysis are presented in Table 6.

**Table 6:** Bootstrapped analysis of indirect effects.

	Path	Effect size	Boot SE	Boot LLCI	Boot ULCI
Direct effect	Self-esteem → appearance anxiety	−0.54	0.05	−0.65	−0.45
Indirect effects	Self-esteem → social media use → appearance anxiety	−0.02	0.01	−0.06	−0.01
	Self-esteem → upward social comparison → appearance anxiety	−0.06	0.02	−0.11	−0.03
	Self-esteem → social media use → upward social comparison → appearance anxiety	−0.02	0.01	−0.04	−0.01
Total effect	Self-esteem → appearance anxiety	−0.65	0.05	−0.76	−0.54

The total effect of self-esteem on appearance anxiety was −0.65 and the direct effect was −0.54. Social media use and upward social comparison both partially mediated the association between self-esteem and appearance anxiety, with an indirect effect of-0.11 that accounted for 16.3% of the total effect. The bootstrapped 95% confidence intervals for all three indirect pathways did not contain 0, indicating that the indirect effects were statistically significant.

### 3.4 Gender differences

We employed multiple-group analysis to examine gender differences in associations between the study variables. We established a constrained model with equal factor loadings, measurement and structural weights, covariance, and residuals between the two genders. Compared with the unconstrained model, the constrained model showed significant differences (∆*χ*^2^ = 124.80, ∆df = 70, *p* < 0.001), indicating the two models differed.

Table 7 shows that every path was significant for women but that the association between social media use and appearance anxiety was not significant for men (*p* > 0.05). This suggests that upward social comparison did not trigger appearance anxiety in the male participants in this sample.

**Table 7:** Multiple group analysis by gender.

Path	Male				Female			
	Estimate	SE	CR	*p*	Estimate	SE	CR	*p*
Self-esteem → social media use	−0.15	0.07	−1.97	0.050	−0.18	0.071	−2.63	0.009
Social media use → upward social comparison	0.62	0.07	6.91	^***^	0.39	0.061	5.60	^***^
Self-esteem → upward social comparison	−0.21	0.05	−3.29	0.001	−0.37	0.059	−5.56	^***^
Upward social comparison → appearance anxiety	0.11	0.08	1.45	0.147	0.30	0.062	4.78	^***^
Self-esteem → appearance anxiety	−0.66	0.07	−7.59	^***^	−0.56	0.058	−8.54	^***^
Social media use → appearance anxiety	0.19	0.06	2.53	0.011	0.16	0.045	3.14	0.002

## 4 Conclusion

We employed structural equation modeling in this study to investigate the association between self-esteem and appearance anxiety and whether social media use and upward social comparison helped explain this association. We found a significant negative association between self-esteem and appearance anxiety, which was partially explained by heightened social media use and a tendency to make upward social comparisons. An assessment of gender differences revealed that upward social comparison was not linked to appearance anxiety in males. The findings have implications for developing interventions for appearance anxiety and highlight the importance for researchers to examine gender differences in appearance-related anxiety issues.

## 5 Discussion

This study aimed to explore the association between self-esteem and appearance anxiety and assess whether social media use and upward social comparison help explain any observed association. Drawing on social comparison theory and using multi-group analysis, we explored potential direct and indirect associations among the core variables, as well as gender-specific patterns in these associations. The findings offer theoretical and practical insights into appearance-related concerns in digital contexts.

### 5.1 Self-esteem and appearance anxiety

We found a significant negative relation between self-esteem and appearance anxiety, consistent with previous findings (Kostanski and Gullone, 1998; Mellor et al., 2010; Adams et al., 2017). Self-esteem reflects the acknowledgement of one’s worth and trust in one’s abilities (Mecca et al., 1989). Heightened self-esteem can help mitigate the negative effect of factors that contribute to appearance anxiety, such as body dissatisfaction, fear of negative judgment, and perceived imperfections (Hart et al., 2008; Levinson et al., 2013). Individuals with low self-esteem show heightened cortisol in response to rejection, make more negative self-evaluations, and tend toward self-blaming attributions (Ford and Collins, 2010). They also have more pessimistic and cautious outlooks compared to those with higher self-esteem (Stinson and Fisher, 2020).

Previous research suggests that bullying (Copeland et al., 2015; Costa et al., 2015) and weight-related teasing (Chen et al., 2019; Dakanalis et al., 2017) can contribute to body dissatisfaction and cause victims to initiate dietary restrictions, suggesting low self-esteem as a mechanism linking painful social experiences with appearance anxiety. A study of adolescents by Fowler et al. (2021) found that childhood experiences of being bullied can lead to lower physical self-esteem. Thus, improving self-esteem may be a feasible strategy to reduce appearance anxiety.

### 5.2 Potential indirect associations via social media use and upward social comparison

Social media use helped to explain the relation between self-esteem and appearance anxiety. Self-esteem was found to have a negative association with social media use, which in turn showed a positive association with appearance anxiety. This aligns with prior research indicating that individuals with low self-esteem use social media more frequently as they perceive it to be a safer communication platform where they can avoid psychological risks. Communication is a form of self-exposure, and such exposure can be risky. Face-to-face communication can be overlooked, rejected, or perceived poorly by others (Andreassen et al., 2017; Forest and Wood, 2012). Moreover, individuals with low self-esteem often experience insecurity, which can negatively impact relationship satisfaction and happiness (Erol and Orth, 2017; Sciangula and Morry, 2009), leading to an increase in compensatory internet use (Kardefelt-Winther, 2014). Additionally, exposure to idealized beauty images on social media can cause individuals to internalize unrealistic beauty standards, contributing to body dissatisfaction and appearance anxiety (Groesz et al., 2002; Vandenbosch and Eggermont, 2012).

Upward social comparison also helped explain the relation between self-esteem and appearance anxiety, with self-esteem negatively associated with upward social comparison, which was positively associated with appearance anxiety. Individuals with low self-esteem possibly make more upward social comparisons due to low self-confidence, a reluctance to believe they are better than others, or a quest for improvement (Kernis et al., 1993; Ybema and Buunk, 1993). Such comparisons can cause feelings of deprivation, reduced well-being, and negative self-perceptions, contributing to body dissatisfaction and appearance anxiety (Park, 2007; Schmuck et al., 2019; Vogel et al., 2014).

Social media use and upward social comparison were found to sequentially mediate the association between self-esteem and appearance anxiety. This aligns with previous findings (Lup et al., 2015; Verduyn et al., 2017; Vogel et al., 2014). Social media platforms prompt constant social comparison, as users encounter a barrage of idealized images. In our proposed path of associations, self-esteem is linked to social media use, which increases upward social comparison, leading to appearance anxiety. A study by Midgley et al. (2021) showed a similar pattern of findings, as students with lower self-esteem were found to engage in more extreme upward comparisons, particularly related to appearance, while browsing social media, leading to a significant decline in self-evaluation.

One possible explanation for the indirect association between social media use and appearance anxiety lies in the curated and idealized content typically found on these platforms. Drawing on self-presentation theory (Goffman, 1959), individuals are motivated to present idealized versions of themselves online, thereby shaping unrealistic beauty standards that viewers may internalize. This idealization is not only driven by personal aspiration, but also by perceived audience expectations and social norms embedded in digital interaction.

Empirical studies have shown that exposure to these curated portrayals can increase body dissatisfaction and appearance-related anxiety, primarily through the internalization of unattainable beauty ideals (Fardouly and Vartanian, 2016; Tiggemann and Slater, 2013). The visual and evaluative architecture of social media—centered on images, likes, and comments—further amplifies this effect by facilitating appearance-focused comparison and reinforcing normative beauty expectations (Vogel et al., 2014). This creates a self-perpetuating cycle of comparison and self-monitoring. Importantly, individuals with low self-esteem may be especially vulnerable to these processes. According to sociometer theory (Leary and Baumeister, 2000), they are more dependent on external validation for self-worth, making them more susceptible to the psychological impact of idealized comparisons and negative feedback. Such individuals may turn to social media as a safer space for interaction, yet paradoxically expose themselves to increased anxiety through intensified exposure and internalization of others’ curated self-presentations (Forest and Wood, 2012; Kardefelt-Winther, 2014).

Taken together, these mechanisms help explain why heavy social media use—particularly among those with low self-esteem—can be associated with elevated levels of appearance anxiety.

### 5.3 Gender differences

We found no significant correlation between upward social comparison and appearance anxiety in men. This suggests upward social comparisons are more closely linked with appearance anxiety among women. This difference may be attributed to increased concerns about appearance in women, which may lead to more frequent upward social comparisons related to appearance and subsequent negative emotions such as anxiety. Girls are more likely to engage in physical monitoring than boys, indicating a greater focus on appearance (Lindberg et al., 2006). Fardouly et al. (2017) found that female college students commonly made social comparisons based on appearance in their daily lives and that these were usually upward comparisons.

Men and women may respond differently to upward social comparisons. Men are more adept at using problem-centered coping strategies to manage stress (Watson and Blanchard-Fields, 1998). When faced with frustration and feelings of inadequacy due to upward social comparison, men may focus more on constructive actions to improve themselves and thus experience less anxiety. Indeed, Weiguo et al. (2022) found in a study of high school students that females were more likely than males to express anxiety and depression and unconsciously lower their self-evaluations in response to upward social comparisons.

This gender difference can be further understood from the perspectives of gender socialization and objectification theory. Women are often socialized to value appearance more heavily, and their self-worth is more likely to be tied to body image and attractiveness (Fredrickson and Roberts, 1997). As a result, women are more susceptible to internalizing societal beauty standards and engaging in appearance-related self-surveillance (Myers and Crowther, 2009).

In contrast, men are more frequently evaluated based on competence, achievement, and social dominance. Their exposure to media imagery often emphasizes performance and strength rather than aesthetic beauty (Myers and Biocca, 1992). Thus, upward comparison based on appearance may have less emotional impact on male users. These findings are consistent with prior literature suggesting that women engage in more frequent and emotionally impactful appearance comparisons than men, especially in digital environments (Moradi and Huang, 2008). This may explain the gender-specific pathways observed in this study.

The gender differences observed indicate that upward social comparison is associated with appearance anxiety in women but not men. This underscores the importance of assessing the detrimental effects of upward social comparisons on women’s appearance-related anxiety and highlights the need for further research on gender differences in appearance-related issues.

### 5.4 Theoretical and practical contributions

The findings of this study contribute to current study in several ways. First, by drawing upon social comparison theory, the study offers a nuanced understanding of how self-esteem, social media use, and upward social comparison interact to predict appearance anxiety, highlighting the dynamic pathway through which individuals’ self-worth is threatened in digital social environments.

Second, the discovery of gender-specific pathways offers new insights into how appearance anxiety develops differently for men and women. These findings support existing theories of gendered self-objectification and extend them to the social media domain, where image-centric interactions intensify gendered psychological vulnerabilities.

Finally, this study focuses on Chinese college students, a group underrepresented in academic research yet highly active on social media platforms within the Chinese digital ecosystem. In collectivist societies like China, individuals may be particularly sensitive to socially shared standards of appearance, as personal image is often perceived to reflect not just individual identity but also group harmony and social expectation (Hu, 1944; Triandis, 1995; Jackson and Chen, 2008b). Previous studies have shown that Chinese youth are highly concerned with body image and are more susceptible to body dissatisfaction resulting from social media exposure (Jackson and Chen, 2008a; Chen et al., 2007). The appearance-related pressures they face are shaped not only by local cultural ideals such as “thinness” and “fair skin,” but also by Westernized standards of “fit” and “healthy beauty” prevalent on global social platforms (Jackson and Chen, 2008a; Swami, 2015). As a result, their social media comparison behaviors and emotional reactions reflect a hybridized cultural pattern. This study extends the largely Western-centered literature on appearance anxiety and responds to ongoing concerns about cultural bias in body image research (Swami et al., 2010). It offers insights toward building a globally inclusive theoretical framework.

In addition to its theoretical implications, this study also suggests two avenues for practical intervention. First, enhancing self-esteem may help reduce vulnerability to appearance-related distress. Prior research has shown that practices designed to enhance self-esteem, such as engagement in sports (Bang et al., 2020), mindfulness training (Yüksel Doğan and Metin, 2023), and art therapy (Yücesan and Şendurur, 2018), can buffer the emotional effects of appearance-based comparisons. Second, interventions targeting social media use and comparison behavior, such as media literacy programs, digital detox practices, and appearance comparison reappraisal training, may reduce individuals’ exposure to unrealistic appearance standards and weaken the internalization of idealized portrayals. These strategies offer promising pathways for school-based or clinical interventions aimed at mitigating appearance anxiety in the digital age.

### 5.5 Limitations and future directions

This study had several limitations. The research was cross-sectional, and as such we are not able to outline causal relations between variables. Future research could employ longitudinal methods and experimental designs to establish causal relations. Secondly, the AVE for the social media use construct was slightly below the recommended threshold, suggesting that future research may consider further refining the measurement of this construct. In addition, the majority of participants were from two provinces in China. Appearance anxiety may vary across regions and by economic development. Future studies could explore these variables in more diverse samples or compare findings across different geographic regions. Meanwhile, social media use was assessed using a usage intensity approach in our study, which reflects the general level of engagement and emotional connection to social media platforms. While this method captures users’ overall involvement, it does not distinguish between specific purposes of use (e.g., entertainment, information-seeking, or self-presentation). Prior research suggests that users may engage with different social media platforms for different purposes and in different ways (Ku et al., 2013). For example, Weibo is primarily used for content gratification such as information acquisition and sharing, whereas WeChat is more often used for social gratification, including private networking and convenient communication (Gan and Wang, 2015). Future research may benefit from incorporating multidimensional measures to explore purpose-specific variations in social media engagement. In addition, the present study did not account for certain potential confounding variables, such as body mass index (BMI), socioeconomic status (SES), and prior mental health conditions. These factors may have influenced both self-esteem and appearance anxiety, and their omission may limit the precision of the estimated associations. Future studies are encouraged to include such covariates to enhance the robustness of model testing and better isolate the effects of key psychological constructs. Finally, due to the self-report and cross-sectional nature of the data, future studies should incorporate additional procedural and statistical remedies, such as reverse-worded items, temporal separation of measurement, or latent method factor analysis, to more rigorously address CMB concerns.

## Data Availability

The raw data supporting the conclusions of this article will be made available by the authors, without undue reservation.
